# Effectiveness of “tumor necrosis factor inhibitors” in monogenic hereditary recurrent fevers in children and adolescents: a systematic review

**DOI:** 10.3389/fimmu.2025.1710180

**Published:** 2025-11-17

**Authors:** Rim Dhahri, Soumaya Boussaid, Lobna Ben Ammar, Insaf Fenniche, Hiba Ben Ayed, Safa Rahmouni, Khaoula Zouaoui, Sonia Rekik, Khalil Amri, Hela Sahli, Imène Gharsallah

**Affiliations:** 1 Rheumatology Department, Military Hospital of Tunis, Tunis, Tunisia; 2 Faculty of Medicine of Tunis, University Tunis el Manar, Tunis, Tunisia; 3 Rheumatology Department, La Rabta Hospital, Tunis, Tunisia; 4 Orthopedics Department, Military Hospital of Tunis, Tunis, Tunisia

**Keywords:** monogenic, Kmonogenic hereditary recurrent fevers, TNF inhibitors, pediatric rheumatology, tumor necrosis factor inhibitors, systematic review

## Abstract

**Introduction:**

This systematic review aims to evaluate the clinical effectiveness of tumor necrosis factor (TNF) inhibitors in treating monogenic hereditary recurrent fevers (HRFs) in children and adolescents.

**Methods:**

We conducted a comprehensive literature search across MEDLINE, EMBASE, and Scopus up to May 2024, including case reports, case series, and observational studies involving pediatric patients with HRFs treated with TNF inhibitors. Articles were screened and selected based on PRISMA guidelines.

**Results:**

Eleven pediatric cases were identified from ten studies, including patients with FMF (n=2), MKD/MKD (n=5), TRAPS (n=2), and CAPS (n=2). Etanercept was the most frequently used TNF inhibitor (10/11 cases), and infliximab was used in one FMF case. Follow-up duration ranged from 3 months to 4 years. Clinical responses varied: full remission in TRAPS cases; partial improvement in some MKD and CAPS cases; and no significant effect in several FMF and MKD/MKD patients. Etanercept showed the best outcomes in TRAPS, while responses in CAPS and MKD/MKD were inconsistent.

**Clinical trial registration:**

TNFi may offer a therapeutic option for selected pediatric HRF cases, particularly colchicine-resistant FMF with articular symptoms or where IL-1 blockers are unavailable. However, their efficacy appears limited and variable across HRF subtypes. Larger studies are needed to better define the role of TNF inhibitors in pediatric HRFs.

## Introduction

Systemic auto-inflammatory diseases (SAIDs) are a heterogeneous group of disorders now known to be caused by disturbances in the inflammasome pathway (1, 2). Their clinical features result from excessive activation of the innate immune system, leading to recurrent inflammatory attacks separated by periods of remission. Some SAIDs are monogenic and are collectively referred to as *Hereditary Recurrent Fevers* (HRFs), including Familial Mediterranean Fever (FMF), Tumor necrosis factor Receptor-Associated Periodic fever Syndrome (TRAPS), Mevalonate Kinase Deficiency (MKD), and Cryopyrin-Associated Periodic Syndromes (CAPS) (3–5).

Diagnosis has been facilitated by the new EUROFEVER/PRINTO classification criteria, which combine clinical manifestations and genotypes with good accuracy, sensitivity, and specificity (6). However, therapeutic management remains less consensual due to their rarity. Although IL-1 inhibitors (e.g., anakinra, canakinumab) are approved and effective for many HRFs (7, 8), TNF inhibitors (TNFi) have occasionally been considered as alternatives, particularly when IL-1 therapy is unavailable, poorly tolerated, or when articular involvement predominates (9–11). This review investigates the available evidence supporting such uses in pediatric patients.

## Methods

We included case reports, case series, and observational studies reporting on pediatric patients (<18 years) with monogenic hereditary recurrent fevers (HRFs) treated with TNFi. Outcomes evaluated included clinical remission, reduction in flare frequency, normalization of inflammatory markers, and follow-up duration.

### Search strategy

We used a comprehensive search strategy that aimed to be both sensitive and specific, following established methodological recommendations for systematic reviews (12–14). We employed detailed search strategies as deemed appropriate for each database (Medline, EMBASE, and Scopus) until May 2024. An initial search strategy was initiated using the MEDLINE thesaurus and indexing system to identify appropriate MeSH headings and key/text words associated with the terms “Tumor Necrosis Factor inhibitors” OR “Adalimumab” OR “Certolizumab” OR “Etanercept” OR “Golimumab” OR “Infliximab” AND “‘Familial Mediterranean Fever’ OR ‘Hereditary Recurrent Fever’ OR ‘Tumour Necrosis Factor Receptor-Associated Periodic Fever Syndrome’ OR ‘Mevalonate Kinase Deficiency’ OR ‘Cryopyrin Associated Periodic Syndromes’.”

This search strategy was adapted for each database as necessary. The references of papers and review articles were also manually checked to ensure inclusion of studies not retrieved through the computerized search method (15, 16).

### Inclusion criteria

Inclusion and exclusion criteria were predefined according to PRISMA recommendations to ensure methodological consistency (12–14).

The following studies were included:

1. Studies including pediatric patients (<18 years) with Hereditary Recurrent Fevers (HRFs).
2. Prospective and retrospective studies assessing the impact of TNFi use on disease outcomes.

Study types included observational (cohort, case-control, cross-sectional) and experimental designs (randomized controlled trials, controlled clinical trials), as well as case reports, case series, and conference abstracts reporting clinical, serological, or therapeutic features of HRFs.

### Exclusion criteria

Studies in languages other than English, review articles, animal models, commentaries, editorials, questionnaire studies, duplicates, and papers not relevant to the topic were excluded.

### Stages in the literature search

The protocol was finalized in April 2024. The various stages of this literature search were summarized using the Preferred Reporting of Systematic Reviews and Meta-Analysis (PRISMA) flow chart to visualize the processes and findings of the review (12).

### Classification of result resources and data extraction

Data extraction sheets and tables were developed and tailored to the included resources according to Cochrane Handbook recommendations (17, 18).

### Study selection

**Figure 1:**
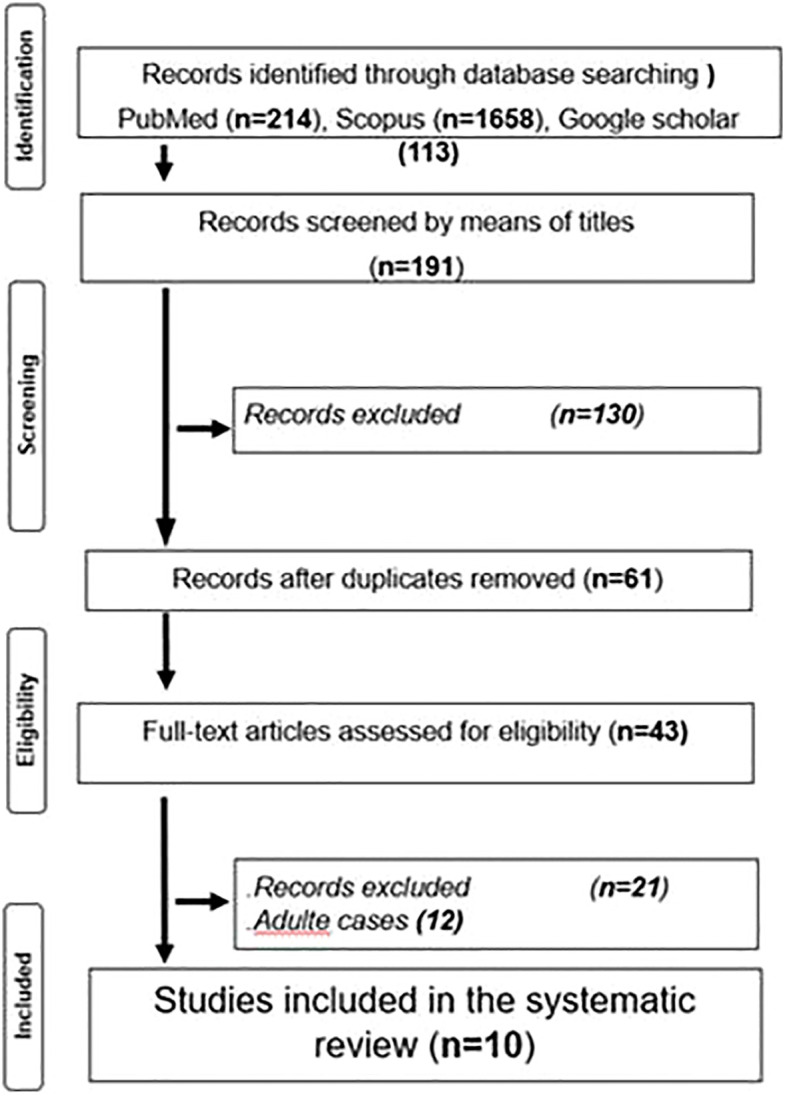
Flow diagram showing the number of records identified, screened and included in the review, as per PRISMA 2020 guidelines.

Titles and abstracts were screened and independently assessed for eligibility by two reviewers (RD and SB); conflicts were resolved by SM. Full-text papers were evaluated in duplicate by RD and SB. Any disagreement regarding eligibility was resolved by discussion with a third reviewer (SM). We extracted the following information from all eligible studies: (1) year of publication, (2) country, (3) study design, (4) sample size, (5) demographics, (6) type of autoinflammatory disease, (7) age at onset, (8) main disease features, (9) episode duration, (10) number of episodes/year, (11) TNF blockers studied, (12) therapeutic response, and (13) duration of follow-up. The PRISMA flow diagram (**Figure 1**) summarizes the study selection process.

## Results

The initial search yielded 1985 papers. After title screens, 191 Records were identified through database searching; PubMed (n=52), Scopus (n=26), and Google Scholar (113). Then after abstract reading, 130 papers were excluded, and after duplicate removal, only 61 articles were retained. After full-text reading and authors’ concertation, only 21 articles were assessed for eligibility. Finally, after adult cases’ exclusion, only 9 cases were retained.

### Main results

A total of 11 pediatric cases from 10 studies were included (9–11, 19–25). These involved patients diagnosed with FMF (n=2), MKD (n=5), TRAPS (n=2), and CAPS (n=2).

Etanercept was the most frequently used TNF inhibitor (10/11 cases), with infliximab administered in one FMF case. Follow-up ranged from 3 months to 4 years.

In the CAPS cases, specific subtypes were not clearly defined; however, clinical features suggested that one patient likely had Familial Cold Autoinflammatory Syndrome (FCAS), while the other had features suggestive of Neonatal-Onset Multisystem Inflammatory Disease (NOMID), including neurological symptoms such as papilledema. Etanercept was administered in 10 cases, though dosing regimens varied across studies: some used 0.4 mg/kg twice weekly, while others reported different frequencies or did not specify dosage. Infliximab was used in one FMF case (26). Follow-up durations, when reported, ranged from 3 months to 4 years, with rapid improvement observed in 4 cases. However, in one MKD/MKD case, recurrent episodes persisted despite combination therapy with anakinra and colchicine, with minor attacks occurring every 4–6 weeks (22). Another MKD/MKD case showed continued relapses until 4 months of treatment, after which attack frequency and severity declined (20). In contrast, one MKD/MKD case did not respond significantly to etanercept (23), and both CAPS cases showed only partial responses. Therapeutic outcomes varied by disease: Complete clinical and biological remissions were achieved in both TRAPS cases, with normalization of inflammatory markers (ESR, CRP, and serum amyloid A) and complete resolution of symptoms under etanercept therapy (20, 23). This consistent response contrasts with the more variable outcomes observed in other HRF subtypes, emphasizing the particular sensitivity of TRAPS to TNF inhibition. partial responses in three cases (one MKD/MKD and two CAPS (26)); and no significant response in six cases, including FMF and three MKD/MKD cases (21, 23). In one FMF report, outcome data were not provided. The lack of precise CAPS subtype classification (e.g., FCAS, Muckle-Wells syndrome, or NOMID) further limited interpretation of treatment responses. The results are summarized in **Table 1**.

**Table 1:** Efficacy of TNFi in HRDs.

Authors/year of publication/country	Type of auto-inflammatory disease	Study design/TNF inhibitor studied	Cases characteristics (age(year)/n/gender)	Age at disease onset	Main disease features	Therapeutic response	Duration of follow up
Kara Eroglu 2015 Turkey (11)	FMF	single-center retrospective case series/Etanercept	14 patients/13.2 ± 6.8 years (2–24 years)	NM	Colchicine Resistant (0.03–0.06 mg/kg/day)/Recurrent protracted Abdominal pain/Fever/Arthritis	Etanercept continued for median 7 months	Mean 7.43 ± 4.6 years (2–16 years).
2. Hua 2023 China (19)	TRAPS	Case Report/Etanercept	16 Y M	NM	the patient was in the intermittent phase of disease episodes and did not show significant symptoms.	significant remission of clinical symptoms and no disease relapse	3 months
3.Kisla Ekinci 2022 Turkey (10)	periodic fever	Case report/monthly intravenous immunoglobulin replacement and weekly subcutaneous etanercept	15 month M	15 month	Sideroblastic Anemia with B Cell Immunodeficiency, Periodic Fevers, and Developmental Delay Syndrome treated with colchicine	A rapid resolution of fever episodes and infections occurred after initiation of this treatment regimen	NM
4.Yuksel 2006 Turkey (9)	FMF Amyloidosis Protracted arthritis	case report/Infliximab 3 mg/kg at 0, 2d, 6, and 8 weeks	12 F	NM	Bloody diarrhea, vomiting, massive proteinuria hypoalbuminemia, knee and ankle arthritis	improvement in massive proteinuria and hypoalbuminemia, full recovery of GIS symptoms and arthritis	22 month
5.Shendi 2009 UK (20)	MKD	case report/10 mg of etanercept was given twice weekly for 9 weeks	10Y F	11 months	recurrent febrile episodes partially	she had fortnightly episodes,	NM
6.Takada 2003 Albania (21)	MKD	case report/etanercept 0.4mg/kg SC twice a week	10 Y F	6 weeks		first: there was no improvement, then, the number of MKD attacks and their severity were decreased.	4 years
	MKD	case report/etanercept 0.4mg/kg SC twice a week	NM	3 day of life		The number of MKD attacks and their severity were decreased	NM
7.Topaloğlu 2008 Turkey (22)	MKD	case report/etanercept with a dose of 0.8 mg/kg per week	20 months M	20 Month old	colchicine and simvastatin The response was partial	great.improvement of both attacks and acute-phase response . persistent massive hepatosplenomegaly.	2 years
8.F Marchetti 2004 Italy (23)	MKD	Case report/etanercept (12.5 mg/dose, 2 days for weeks)	4 years M	6 months	steroid treatment was started at the beginning of the episodes of fever, with partial evidence in the reduction of symptoms but not in the recurrence of the episodes	No significant effects were reported for all parameters considered. 5 episodes of fever with abdominal pain and cervical lymphadenopathy	3 months
9.H Morbach 2009 USA (24)	TRAPS	case report/etanercept 0.4 mg/kg subcutaneously twice a week	16 Years F	NM	periodic erythema and myositis/fasciitis. while no fever episodes. A delay of puberty with amenorrhea and a short stature	.a gain of weight and a rapid development of puberty.Episodes of focal myositis and erythema decreased in intensity and in frequency.ESR, CRP, and serum amyloid A were normal.anemia improved .IgG titers remained elevated .significant clinical improvement	5 years
10.Tahghighi 2022 Iran (25)	CAPS	1 case reports/etanercept at a dose of 0.8 mg/kg per week subcutaneously	1 Y M	NM	Since birth	.joint involvement improved. .a mild effusion in knee during treatment with etanercept. urticarial-like rash had decreased .At the age of 4, he developed eyelids’ swelling and erythema	6 months
	CAPS	etanercept at a dose of 0.8 mg/kg per week subcutaneously	2 Y F	recurrent purpuric urticarial rash since three months		fever was relatively controlled, but the rash did not respond .Relapse occurred after four months of treatment	6 months

CAPS, Cryopyrin-Associated Periodic Syndromes; FMF, Familial Mediterranean Fever; MKD, Mevalonate Kinase Deficiency; PFAPA, Periodic Fever Aphthosis, Pharyngitis and Adenitis; TRAPS, Tumor Necrosis Factor Receptor-Associated Periodic Fever Syndrome; ESR, Erythrocyte Sedimentation Rate; CRP, C Reactive Protein; TNF, Tumor Necrosis Factor.

## Discussion

Since HRFs are a heterogenous group of AIDs with clinical, biological and genetic particularities. We treated each HRF a part.

### Familial Mediterranean fever

FMF is the most common monogenic autoinflammatory disease (27). It is an autosomal recessive genetic disorder associated with mutations in the MEFV gene located on chromosome 16, which were identified in 1997 (28).

This disease is more common in non-Ashkenazi Jewish, Arab, Armenian and Turkish communities (29).

The MEFV gene encodes the pyrin protein, whose excessive activation leads to hypersecretion of interleukin 1 (29).

Symptoms and progression of the disease

The first symptoms appear before the age of 20 in 90% of cases, with an average age of 4, and before the age of 1 in 10% of cases. Acute attacks generally last between 1 and 4 days (30).

Clinically, the disease is characterized by recurrent episodes of fever appearing in childhood, accompanied mainly by abdominal pain, serositis, joint symptoms (arthralgia and/or arthritis) and a biological inflammatory (31).Joint involvement is the second most common manifestation after abdominal pain (31) Treatments and complications.

The usual treatment is based on daily, lifelong colchicine to prevent the most serious chronic complication, AA amyloidosis.

The EUROFEVER registry has documented 121 patients with FMF, with a median age of 11.6 years at registration and 3 years at disease onset, all treated with colchicine as first-line therapy (32).

According to a review by (33), colchicine induces complete remission in two-thirds of pediatric patients and prevents AA amyloidosis. However, 5–10% of patients do not respond to treatment or experience severe side effects. Alternative therapies are therefore being explored (34, 35).

Although the mechanism of action of TNF- in the pathogenesis of FMF is not fully understood, drugs targeting this protein have been used for about ten years in patients resistant to colchicine. Their efficacy remains a subject of debate (34, 36). Indeed, the use of TNFi in FMF is mainly justified in patients with predominant articular involvement, such as chronic arthritis or spondyloarthritis, rather than in those with systemic febrile attacks. The established efficacy of TNF blockade in other chronic arthritides, such as rheumatoid arthritis and spondyloarthritis, suggests a shared inflammatory pathway that may also contribute to persistent FMF-related arthritis. This mechanism differs from the acute febrile flares primarily mediated by IL-1 activation. Therefore, TNFi may act downstream of IL-1 to modulate chronic joint inflammation rather than suppressing inflammasome-driven fever episodes.

It has been established that the symptoms of FMF result from abnormal release of IL-1. Over the past ten years, biotherapies targeting IL-1 have been shown to be effective in patients who are resistant or intolerant to colchicine (11, 33, 35). The effects of IL-1 vary depending on the cell type and can perpetuate recurrent inflammatory responses via the induction of factors such as tumor necrosis factor (TNF)-α, nitric oxide synthase and prostaglandin E2 (36).

Role of biotherapies and clinical recommendations

TNF blockade with etanercept, infliximab or adalimumab has been studied, particularly in patients with persistent arthritis, with results showing partial or complete remission (37).

For example, in our review, the infliximab-treated FMF case (**Table 1**) achieved complete remission of articular symptoms, reinforcing the hypothesis of a TNF-mediated inflammatory pathway specifically driving chronic joint manifestations, distinct from the IL-1-induced febrile phenotype.

Infliximab, a monoclonal anti-TNF antibody, has been shown to be effective in controlling FMF attacks and improving symptoms. According to several case reports and data from the Eurofever registry, anti-TNF antibodies are a good option for patients who do not respond to colchicine (26).

In November 2017, the Haute Autorité de Santé published recommendations for the use of canakinumab, which should be reserved for forms of FMF that are resistant to colchicine and prescribed by a reference or competence center (38).

Anti-TNF agents may also be an alternative to colchicine, especially in patients with FMF with predominant joint involvement (persistent peripheral arthritis or spondyloarthritis) or associated inflammatory bowel disease (Crohn’s disease) (35, 37, 39).

Langevitz (40) identified 11 cases meeting the criteria for seronegative spondyloarthropathy (SNSA) in their study of 3,000 patients, suggesting that this condition could be one of the forms of musculoskeletal involvement and develop despite treatment with colchicine (41). also suggested a link between ankylosing spondylitis (AS) and FMF. Cases of FMF treated with a tumor necrosis factor alpha (TNF-α) inhibitor have been reported (36, 41, 44].

In general, with regard to biotherapies for FMF, it is recommended that the indication be made by a reference center, such as CEREMAIA (reference center for autoinflammatory diseases and inflammatory amyloidosis), during its monthly multidisciplinary consultation meeting (38).

Etanercept was used before anti-IL1 agents. Positive responses have been reported in some cases, particularly in patients with chronic arthritis (42). However, in our own patients, etanercept did not produce a lasting response (11).

TNF Receptor-Associated Periodic Syndrome (TRAPS)

TRAPS ranks as the second most common HRF. This monogenic, autosomal dominant disease is caused by mutations in the TNFRSF1A gene, leading to dysfunction of the tumor necrosis factor receptor (37).

It can occur at any age, including infants and young children (27).Typical symptoms include fever attacks, polyseritis, and joint and skin involvement (43).Data from the EUROFEVER registry show significant improvement with TNF inhibitors. Etanercept, for example, was beneficial in 32 of 37 patients, but only 11 (30%) achieved complete remission (8).

In the two clinical cases we reviewed, the patients responded perfectly to etanercept (20, 24). Both TRAPS cases demonstrated complete clinical and biological remission, supporting the consistent efficacy of TNF blockade in this condition. This observation aligns with previous registry data showing that TRAPS, unlike FMF, MKD, or CAPS, may respond particularly well to TNFi therapy. While IL-1 inhibitors remain first-line treatment, TNF inhibition appears to be a valid and effective second-line therapeutic alternative in TRAPS, especially when IL-1 blockade is not tolerated or available.

There are no reported data regarding the use of other TNFi in this setting.

### Mevalonate kinase deficiency syndrome

Formerly known as hyperimmunoglobulinemia D, MKD syndrome is a rare autosomal recessive autoinflammatory disease caused by mutations in the gene encoding MVK.

Symptoms typically appear before the first birthday. Diagnosis is made by confirming the presence of a pathological MVK genotype and at least one of the following signs: gastrointestinal disturbances, cervical lymphadenitis, or aphthous stomatitis (43).

The EUROFEVER registry indicates that etanercept was effective in 11 of 17 treated patients (65%), although only one complete remission was documented. Information specific to the pediatric population was not available (8).

### Cryopyrin-associated periodic febrile syndromes

CAPS encompasses several autoinflammatory diseases caused by mutations in the NLRP3 gene (previously called CIAS1) on chromosome 1. Familial cold autoinflammatory syndrome and neonatal multisystem inflammatory disease (NOMID) are the most extreme forms of this pathological spectrum.

In children, symptoms often begin early and include fever, skin lesions, and joint involvement (28).

NLRP3 mutations trigger excessive inflammasome activation, which explains the use of IL-1-targeted therapies. In the two cases analyzed, TNF-α inhibitors relieved mild symptoms (fever, rash) but did not improve more severe complications such as papilledema or arthritis. Given the rarity of this disease, few clinical data are available (26).

In our review, TNFi produced only partial or transient benefits in CAPS cases, with improvement in mild symptoms such as fever or rash, but no effect on neurological involvement or chronic inflammation. Notably, one case presented features consistent with the severe NOMID phenotype, which did not respond to etanercept. This lack of efficacy in the most severe CAPS subtype highlights a major limitation of TNFi use in these disorders. The incomplete specification of CAPS subtypes in the literature (FCAS vs. NOMID) also limits interpretation, but available evidence suggests that TNF blockade is insufficient to control inflammasome-driven inflammation dominated by IL-1 signaling.

This review is limited by the small number of pediatric cases, heterogeneity of study designs, and lack of standardized outcome measures. Most available data are derived from case reports, which limits generalizability. Future multicenter and prospective studies using standardized efficacy endpoints are needed to confirm these findings and define the optimal positioning of TNF inhibitors within HRF therapeutic strategies. In addition, collaborative international registries may help identify specific genetic or clinical predictors of TNFi responsiveness in pediatric HRFs.

## Conclusion

Most of the available data come from isolated cases or small series, making it difficult to accurately quantitatively assess the efficacy of TNFi for HRF, particularly in children.

Nevertheless, published cases suggest that these molecules may constitute a therapeutic option for children and adolescents with colchicine-resistant or intolerant FMF (familial Mediterranean fever), especially in cases of persistent or predominant joint symptoms. For other types of FMF, results remain uncertain.

Due to the availability of more effective and validated anti-IL-1 treatments, TNFi should not be used as first-line therapy. However, in specific situations-such as colchicine-resistant FMF with joint involvement, or limited access to anti-IL-1 agents-they may be considered as a temporary or complementary solution, under strict medical supervision.

TNFi may represent a secondary therapeutic option for selected pediatric patients with hereditary recurrent fevers, particularly for colchicine-resistant FMF with predominant articular manifestations and for TRAPS, in which complete clinical and biological remissions were observed.

While IL-1 inhibition remains the first-line therapy for TRAPS, TNF blockade demonstrated clear efficacy in the reviewed cases, making it a viable second-line option in selected patients. In contrast, responses in MKD and CAPS were partial or inconsistent. Future research should focus on defining biomarkers of TNFi response and on developing multicentric registries to guide personalized treatment strategies in pediatric HRFs.

## Data Availability

The original contributions presented in the study are included in the article/supplementary material, further inquiries can be directed to the corresponding author/s.
